# Investigation of the Formal Pathogenesis of Green Liver Discoloration in Organically Reared Female Bronze Turkeys (*Melleagris gallovapo*)

**DOI:** 10.3390/ani13091558

**Published:** 2023-05-06

**Authors:** Ines Stegmaier, Kerstin Cramer, Larissa Cuta, Gábor Koeller, Christian Visscher, Bernd Reckels, Axel Schoeniger, Maria-Elisabeth Krautwald-Junghanns, Volker Schmidt

**Affiliations:** 1 Clinic for Birds and Reptiles, Faculty of Veterinary Medicine, University of Leipzig, An den Tierkliniken 17, 04103 Leipzig, Germany; 2 Large Animal Clinic Laboratory, Faculty of Veterinary Medicine, University of Leipzig, An den Tierkliniken 11, 04103 Leipzig, Germany; 3 Institute for Animal Nutrition, University of Veterinary Medicine Hannover, Foundation, Bischofsholer Damm 15, 30173 Hannover, Germany; 4 Institute of Physiological Chemistry, Faculty of Veterinary Medicine, University of Leipzig, An den Tierkliniken 1, 04103 Leipzig, Germany

**Keywords:** green liver, Turkey osteomyelitis complex, clinical chemistry, hematology, feed analysis, vitamin E, selenium

## Abstract

**Simple Summary:**

Previous studies indicate that green liver discoloration in fattening turkeys, which has occurred with a high prevalence in organically reared turkeys, is not caused by a certain pathogen but by a variety of factors weakening the immune system. Other than vitamin D_3_ supplementation and feed withdrawal, the influence of the provided feed, including nutrient and energy content, has not been investigated. This study aimed to investigate the state of the immune system and liver function of affected turkey hens and to determine a possible nutritional influence on the pathogenesis. Hematological analyses, clinical chemistry analyses, and determination of vitamin E and selenium liver concentrations at 2 examination dates (70th to 75th and 120th to 127th day of fattening, respectively) as well as feed analyses throughout the whole fattening period were performed. The results indicate a subacute inflammatory process in flocks with the occurrence of green livers at the early fattening stage and acute inflammation in individuals with green livers at the late fattening stage. It is suspected that an inadequate nutrient supply contributes to the development of green liver discoloration, but further research under experimental conditions is needed.

**Abstract:**

Green liver discoloration (GL) in fattening turkeys is suspected to be a multifactorial disease complex with a compromised immune system as the key factor. This study aimed to identify the formal pathogenesis of GL and to investigate possible nutritional influences. A total of 360 Bronze turkey hens out of 10 flocks from 5 fattening farms were necropsied for detection of GL during 2 consecutive trials on 2 examination dates each (70th to 75th and 120th to 127th day of fattening, respectively). At each examination date, hematological and clinical chemistry analyses, as well as determination of vitamin E and selenium concentrations in the liver, were carried out in 6 hens with (if applicable) and 6 hens without GL, representing a total of 130 individuals. Raw nutrient, energy, amino acid, bulk and trace element, and vitamin E and D_3_ concentrations were analyzed in feed samples for each of the five feeding phases during each trial. The results of the hematological analyses, clinical chemistry analyses, and determination of vitamin E and selenium liver concentrations were statistically evaluated between: (i) individuals with and without GL, and (ii) individuals from flocks with and without turkeys with GL. At both fattening stages, the occurrence of GL was characterized by an inflammatory reaction. A subacute inflammatory reaction was detected in the early fattening stage, indicating a viral cause of the disease. In the late fattening stage, acute inflammation indicated a bacterial cause of the disease. The results of the feed sample analyses of the different flocks were generally quite homogeneous. However, the nutrient and energy content of the feed likely contribute to GL pathogenesis.

## 1. Introduction

The occurrence of green livers (GL) in turkeys has been observed for decades, especially in association with arthritis, osteomyelitis, or both, and was first described as green liver syndrome or turkey green-liver osteomyelitis complex (TOC) in the United States [1,2,3,4]. It occurs in fattening turkeys with an increasing frequency from the age of 9 to 10 weeks and seems to be a chronic disease in otherwise often healthy-appearing fattening turkeys [3]. During extensive studies on the health status of conventionally and organically reared turkeys in the Federal Republic of Germany between 2007 and 2017, a noticeable difference in the occurrence of GL between both farming methods was found at slaughter. In organically reared turkeys, 27.7% of the hens (33.2% of Kelly BBB (Broad Breasted Bronze) hens and 24.0% of B.U.T. (British United Turkeys) hens (B.U.T. 6/test product 7 and 9)) and 34.8% of the toms (Kelly BBB) had GL, whereas for conventionally reared turkeys (B.U.T. 6), GL was found in only 4.4% of the hens and 3.8% of the toms [5,6,7]. According to Cullen and Stalker [8], green discoloration of the liver is caused by the accumulation of bile pigment which can be caused by a post or intrahepatic disruption of bile flow. A variety of factors have been taken into consideration as triggering causes for this disorder. The most extensively studied are infectious factors. In the past, different studies reported that 33.0 to 58.3% of turkeys with GL also had so-called TOC lesions [3,9]. Some of the green livers were sterile, but in many of the livers, and especially in simultaneously altered joints or bones, bacteria could be isolated [3,10]. A multifactorial process has been suggested, in which a weakened immune system plays a bigger role than the pathogenicity of certain agents [11]. In a recent study, significant correlations were found between GL and the detection of immunosuppressive turkey hemorrhagic enteritis virus in the early stage of the fattening period and joint/bone lesions in the late stage of the fattening period [12]. Two different pathogeneses of the development of GL were suspected [12].

Bayyari et al. [11] found turkeys with green-liver osteomyelitis complex to have higher heterophil, monocyte, and total white blood cell (WBC) counts and higher total serum protein (TP), uric acid (UA), and urea nitrogen levels as well as lower lymphocyte counts and hemoglobin (Hb), iron, alkaline phosphatase (AP), and gamma-glutamyl transferase (GGT) levels. However, turkeys in this study were considered to be suffering from green-liver osteomyelitis complex if TOC lesions were diagnosed, while it is not stated whether any of them had GL as well [11]. Reference ranges for hematological values in turkeys have been described but vary greatly [13,14,15]. Regarding clinical chemistry parameters, reference ranges specifically for turkeys have been published only for some values [13,15,16,17]. For several parameters, comparative values from studies on turkeys can be found [18,19,20], for others like the albumin to globulin (A:G) ratio, bile acids (BA), glutamate dehydrogenase (GLDH), and low-density lipoprotein (LDL), only published values for other species can be used for comparison [21,22,23,24].

Huff et al. [25] found that the withdrawal of feed, water, or both up to 54 h had no effect on the incidence of GL in Nicholas Large White turkeys. Vitamin D₃ supplementation was proven to significantly reduce the incidence of GL and TOC lesions and to lower heterophil to lymphocyte (H:L) ratios after repeated immunosuppressive treatment by dexamethasone at 5 and 12 weeks of age, respectively [26]. Other than this, no studies have been performed examining the possible nutritional influences on the development of GL. Considering the higher incidence of GL in turkeys reared under organic conditions compared to conventional farming, the feeding regime needs to be taken into consideration as a possible factor in the pathogenesis of GL. It is known that nutrient deficiency and, in some cases, excess can compromise a bird’s immune response [27,28]. Energy and certain substrates, like amino acids, are needed by the immune system to recruit new monocytes and heterophils from bone marrow, for lymphocyte proliferation, and the synthesis of effector and communication molecules [27]. An insufficient protein supply has been shown to cause a reduction in immunocompetence in chickens and turkeys [29,30]. Furthermore, cells of the immune system carry receptors for dietary-regulated hormones, e.g., insulin, glucagon, thyroxin, and catecholamines, which is why the immune system can be influenced by feed intake [27]. Krautwald-Junghanns et al. [6] found a number of deficits in feed samples from organic turkey farms. Compared to feeding recommendations given by the Society of Nutrition Physiology (GfE), insufficient contents of several (semi-)essential amino acids (mainly methionine (Met), cysteine (Cys), and lysine (Lys)) as well as of crude protein (CP) were found in most samples [6,31,32]. Energy levels were insufficient, especially in the later phases of fattening. Calcium (Ca) and phosphorus (P) levels were below recommendations in all analyzed samples throughout all feeding phases, and zinc (Zn) and manganese (Mn) levels in some of them. Most of the feed samples showed slightly increased contents of sodium, chloride, and potassium [6]. In 30% of the samples, selenium (Se) concentrations exceeded maximum legal limits [33]. In 70% of the samples, concentrations were below the detection limit of 0.23 mg/kg original substance (OS) in the formerly applied analytical method [6], so the contents might have been below the recommended level. Chronic deficiency of the antioxidants vitamin E or Se, the latter being a component of glutathione peroxidase (GPX), can negatively impact the immune defense by affecting the function of the antioxidant system [34,35,36]. One of the main storage organs for vitamin E and Se is the liver [36]. Hepatic concentrations of both substances highly depend on the respective feed contents, so published values show a wide range [37,38,39,40]. No reference values have yet been described specifically for organically fed turkeys.

The aim of this study was to identify a possible systemic inflammatory process in the pathogenesis of GL and to determine if the liver function of affected Bronze turkey hens is compromised. In order to work out the formal pathogenesis of GL on flock level, in addition to examining differences between individual turkey hens with and without GL, it was aimed to investigate if significant differences occurred between turkeys from flock samples in which GL was present in at least one hen as opposed to flocks without GL at the respective time point of examination. It was attempted to clarify the possible influence of feed nutrient contents on Bronze turkeys’ immune systems and as such, on the development of GL.

## 2. Materials and Methods

The examinations did not require notification or approval, as in accordance with the German Animal Welfare Act [41].

### 2.1. Animals and Examination Time Points

Based on the selection criteria of a high prevalence of GL within the flocks during the predecessor study [6,7], five organic Bronze turkey fattening farms were selected for investigation. All farms had transitioned the Bronze turkeys’ genetic base from Kelly to Cartier in the last five years. Only hen flocks were chosen for examination to maximize validity, as the GL prevalence in female Bronze turkey flocks had shown greater variance (1.7% to 75.0%) than in the tom flocks (1.7% to 55.0%) in the previous study [6]. Two consecutive trials were included, with the first examination trial starting in September 2020 and ending in March 2021, and the second trial starting in February 2021 and finishing in August 2021. Two flocks from each of the five farms were examined in the early fattening stage between the 70th and 75th day of fattening and shortly before slaughter in the late fattening period between the 120th and 127th day. Due to avian influenza control measures in the area of one of the farms and the SARS-CoV-2 pandemic, two examinations in the late fattening stage had to be canceled. The sample size was calculated based on an average prevalence of GL of 27.7% in organically raised hens (Kelly BBB and B.U.T.) with the lower limit of the 95% confidence interval of 18.5%, as documented in the previous study [6]. Using this lower limit and calculations, according to Cannon and Roe [42], with an allowed maximum herd size of 2500 turkeys [43], a sample size of *n* = 16 for each examination, trial, and flock was required to detect green discolored livers in organically reared hens. Taking into consideration that, on the one hand, a sufficient number of carriers in each sample had to be obtained, and, on the other hand, all turkeys had to be purchased and taken from the food chain, 20 randomly sampled individuals were taken from each flock at each examination date (360 turkeys in total). In some flocks, male turkeys were falsely integrated within the hen flocks (and only noticed during postmortem examination); as with young age, sex is not easily identifiable on the farms. If toms were included in the random sample, these were not further examined and not included in the study. The turkeys were taken to the Clinic for Birds and Reptiles at the University of Leipzig, housed overnight, and provided with feed and water. The next day, stunning (Large Poultry Stunner, Friedr. Dick GmbH & Co. KG, Deizisau, Germany) and euthanasia by exsanguination through a unilateral neck cut severing the carotid artery and the jugular vein were performed. All turkeys were examined for GL and six hens without and (if applicable) six hens with GL were chosen for further examination, which included hematological and clinical chemistry analyses as well as determination of hepatic vitamin E and Se concentrations. Overall, samples were taken from 130 Bronze turkey hens, of which 67 animals were examined at the early and 63 animals at the late fattening stage. 

### 2.2. Data Assessment

#### 2.2.1. Blood Analyses

During the turkeys’ bleeding, blood was collected in EDTA tubes (Sarstedt AG & Co., Nümbrecht, Germany), heparin tubes (Sarstedt AG & Co.), and Eppendorf tubes without coagulant (Eppendorf AG, Hamburg, Germany). The blood in the EDTA and heparin tubes was stored in a cooling box. Blood smears were prepared from native blood and fixed and stained using DiffQuik (RAL Diff-Quik, Siemens Healthcare Diagnostics GmbH, Eschborn, Germany). A total leucocyte count was carried out using the estimation method [44]. Differential blood analysis was performed by counting 100 leucocytes at a magnification of 1000×. Absolute numbers of leucocyte fractions were calculated in ×10^9^ per liter. The EDTA blood was used for the determination of packed cell volume (PCV) and Hb concentration. PCV was determined using a hematocrit centrifuge (Andreas Hettich GmbH & Co. KG, Tuttlingen, Germany). The determination of Hb followed the method described by Kyaw et al. [45]. Eight samples were unusable for PCV and Hb determination due to coagulation.

Albumin (ALB), AP, aspartate aminotransferase (AST), bilirubin (BIL), Ca, cholesterol (CHOL), creatine kinase (CK), GGT, GLDH, urea, lactate dehydrogenase (LDH), LDL, magnesium (Mg), inorganic phosphate (Pi), triglycerides (TG), TP, and UA were analyzed photometrically using the analyzing system Cobas C311 (Roche Diagnostics Deutschland GmbH, Mannheim, Germany) and the associated reagents. The reliability of the measured parameters was tested daily using the intended controls (Roche Diagnostics Deutschland GmbH). Bile acids were determined using the same analyzing system and an appropriate reagents kit (Labor + Technik Eberhard Lehmann GmbH, Berlin, Germany). Analysis of GPX concentration was performed using the Cobas C311 and appropriate test kit (Ransel Randox), as well as the intended controls (Randox Laboratories Ltd., Crumlin, UK). Given that GPX is located in the membrane of erythrocytes, the results are presented related to PCV and Hb in order to compensate for possible changes in erythrocyte numbers or dehydration effects. Eight samples were not eligible for PCV and Hb analysis due to coagulation and were therefore not used for GPX determination. One sample was not suitable for Mg and Ca determination. One sample was not suitable for any clinical chemistry analysis. The globulin concentration (TP minus ALB concentration), A:G ratio, and calcium to phosphate ratio (Ca:Pi) were calculated using the presented results. 

#### 2.2.2. Vitamin E and Selenium Liver Concentration Analysis

Liver tissue samples were taken and stored in a polystyrene box filled with dry ice. At the end of each examination day, the samples were stored at −80 °C until further processing. Determination of the vitamin E liver concentration was achieved by solvent extraction with ethanol, followed by hexane extraction and separation by reversed-phase high-performance liquid chromatography using δ-tocopherol as an internal standard [46,47]. For the determination of the liver Se concentration, the atomic absorption spectrometer Solaar M6 (Thermo Fisher Scientific GmbH, Dreieich, Germany) was used. One gram of homogenized liver tissue was dried at 105 °C in a hot air sterilizer for 24 h, dissolved in 4 mL nitric acid (65% Suprapur, Merck KGaA, Darmstadt, Germany) and incinerated in the microwave digestion system MLS Ethos (MLS Mikrowellen-Labor-Systeme GmbH, Leutkirch, Germany). Afterward, the incinerated liver homogenate was transferred to centrifuge tubes (Corning Falcon 15 mL Falcon-Tubes; Fisher Scientific GmbH, Schwerte, Germany) and filled up to 6 mL with double-distilled water. The homogenate was diluted 1:5 and 20 µL was put in omega cuvettes (Thermo Fisher Scientific GmbH) using an autosampler. The subsequent atomization was performed at 2300 °C. Hepatic Se concentration determination was then achieved by absorbance measurement at an element-specific wavelength (196 nm) using an internal matrix calibration. 

#### 2.2.3. Feed Analysis

In total, 59 feed samples were collected and analyzed. These 59 samples were made up of one sample from every flock for each of the five phase feeds applied (starter feed, phases 1, 2, and 3, and final fattening feed) and some additional samples: one more starter feed and phase 1 sample were collected. In the appropriate two trials, feeds with different textures (granulated and pelletized) were offered in one of the phases each, and both types were analyzed. Furthermore, a retention sample from the manufacturer was analyzed for one starter feed in addition to the sample taken directly from the farm, as some of the first analytical results varied greatly from the other samples. The results from the original and retention samples were very similar; only those from the retention sample were used for statistical analyses. Two farms combined the complete diet with greater amounts of grains in both trials, so the grains were analyzed as well (five samples of oat and one sample of wheat).

Dry matter (DM), crude ash (ASH), CP, crude fat (CFAT), crude fiber (CF), nitrogen-free extracts (NFE), starch, sugar (SU), apparent metabolizable energy corrected by nitrogen (AMEn), amino acids, aspartic acid (Asp), taurine (Tau), threonine (Thr), serine (Ser), glutamic acid (Glu), glycine (Gly), alanine (Ala), valine (Val), Cys, Met, isoleucine (Ile), leucine (Leu), tyrosine (Tyr), phenylalanine (Phe), histidine (His), Lys, arginine (Arg), OH-proline and proline (Pro), bulk elements Ca and P, and trace elements Zn, Mn, and Se as well as vitamins D_3_ and E were analyzed or calculated. This was achieved according to the official methods of the Association of German Agricultural Inspection and Research Institutes (VDLUFA) in the latest updated version [48] at the Institute for Animal Nutrition of the University of Veterinary Medicine Hannover, according to the laboratory, with some modifications which have been described by Leurs et al. [49], except for the AMEn which was calculated according to Commission Regulation (EC) No 152/2009: MJ/kg of ME = 0.1551 × %CP + 0.3431 × %CF + 0.1669 × %starch + 0.1301 × %SU [50]. The vitamin D₃ analysis was performed at Agrolab Lufa GmbH in Kiel, Germany, following a modified version of the official method of VDLUFA lll, 13.8.1. The sample was subjected to alkaline hydrolysis and vitamin D₃ was extracted using petroleum spirit. Using two HPLC methods and UV detection, the crude extract was cleaned and vitamin D₃ and D₂ were quantified. The result was corrected by the recovery rate of the internal standard (vitamin D₂). The weight of the test sample and aliquoting procedure during the different steps of the process were adjusted by the laboratory. The detection limit was 1000 IU/kg OS, i.e., 880 IU/kg feed at 88% DM [51].

In each flock, the five different diets were offered during differing time periods throughout the fattening period. In order to be able to compare the offered nutrient contents with feed recommendations, the fattening period was divided into six supply phases (with some flocks going into a seventh phase) according to the feed recommendation periods by the GfE [31], which include phase 0 (week 1 and 2), phase 1 (week 3 and 4), phase 2 (weeks 5 to 8), phase 3 (weeks 9 to 12), phase 4 (weeks 13 to 16), and phase 5 (weeks 17 to 20). The average energy and nutrient contents offered in each phase were calculated by considering the concentrations offered daily. For some nutrients (CP and all amino acids, CF, Ca, P, and Ca:P), a comparison with the recommendations was only possible up to phase 4, as recommendations for female turkeys are only available for these phases. Recommendations for AMEn, Zn, Mn, Se, and vitamins D₃ and E are given for older turkey hens as well. Comparisons were also made with recommendations for the concentrations of the most important amino acids related to the energy level, as the energy level affects the amount of feed intake [52]. These recommendations were published by Jeroch [53] using the amino acid and energy level recommendations by the GfE [31]. Recommendations by the National Research Council [54] and the latest turkey feeding recommendations by the Polish Academy of Sciences and the Polish Branch of the World’s Poultry Science Association [55] were considered but could not be compared directly as their recommendation periods differ from the ones used by the GfE. All feeding recommendations were developed for heavy, conventionally fattened turkeys, to which the examined Bronze turkeys do not belong. However, feeding recommendations for lighter turkey genetics and organic turkey rearing have not yet been established.

### 2.3. Statistical Analysis

Statistical data analysis was performed using IBM SPSS Statistics for Windows, version 28.0 (IBM SPSS, Armonk, NY, USA). Results were considered significant if the double-sided *p*-value was less or equal to 0.05. Data were examined for normal distribution using the Shapiro–Wilk test. When two groups were compared, the Student’s *t*-test for independent samples was performed in case of a normal distribution and the Mann–Whitney U test if data did not follow a normal distribution. When correlations were calculated, Pearson’s correlation coefficient (in case of a normal distribution for all parameters) or Spearman’s rank correlation coefficient (no normal distribution) were calculated. GGT and bilirubin were not included in the statistical analyses because the concentration of only 7 and 46 (out of 130) samples were above the detection limit, respectively. As age-dependent differences for several parameters have been described [14,17,18], all blood and liver analysis results are shown separately for both examination time points.

## 3. Results and Discussion

GL amongst the examined Bronze turkey hens occurred with an overall prevalence of 8.7% during the early fattening stage (with a range of 0.0% to 68.4% within the different flock samples) and 9.4% in the late fattening stage (with a range of 0.0% to 26.3%; Table 1). Prevalence was thus considerably lower than the 27.7% described for organically reared turkey hens at slaughter by Krautwald-Junghanns et al. [6]. 

### 3.1. Hematological Analysis

Bronze turkey hens with GL showed higher WBC numbers at both examination times as opposed to those without (*p* = 0.011 for the early fattening stage; *p* = 0.007 for the late fattening stage). Lymphocyte counts were higher in the early fattening stage (*p* = 0.048), whereas higher heterophil counts (*p* = 0.025) and higher H:L ratios (*p* = 0.030) were detected in turkeys with versus those without GL in the late fattening stage (Table 2). 

Six Bronze turkey hens showed a marked leukocytosis (>50 × 10^9^/L) with heterophilia (>30 × 10^9^/L), of which five had GL. At the early fattening stage, Bronze turkeys from flock samples with GL showed higher numbers of WBC (*p* = 0.003), heterophil (*p* = 0.010), and basophil granulocytes (*p* ≤ 0.001) as well as monocytes (*p* = 0.034), whereas in the late fattening stage, basophil granulocytes were higher (*p* = 0.041) in turkeys from flock samples with the occurrence of GL compared to those without (Table 3).

The results of the WBC and the differential blood count confirm that stimulation of the immune system of individual Bronze turkey hens affected by GL occurred during both fattening stages. Elevated lymphocyte counts in the early fattening period indicate a viral disease; elevated heterophil counts in the late fattening stage are likely linked to an acute inflammatory disease [56,57]. Furthermore, the stimulation of the immune system seems to have taken place at the herd level when a certain flock was affected by GL early during fattening, at 70 to 75 days.

### 3.2. Clinical Chemistry Analyses

In the clinical chemistry analysis, Bronze turkey hens with GL showed a lower A:G ratio than turkeys without liver alterations on both examination dates (*p* = 0.003 for the early fattening stage; *p* ≤ 0.001 for the late fattening stage; Table 4). Similarly, turkeys from flocks with the occurrence of GL had a lower A:G ratio than animals from non-affected flocks on both examination dates (*p* = 0.039 in the early stage; *p* = 0.001 in the late fattening stage; Table 5).

A decrease in the A:G ratio is considered indicative of acute or chronic inflammation resulting from a rise in alpha-, beta-, and/or gamma-globulins, and often a fall in ALB concentration [58,59]. Besides its role in maintaining osmotic pressure and being a carrier protein, ALB is considered a major antioxidant in the plasma [60,61]. The low A:G ratio originated from a lower ALB concentration in the early fattening stage (*p* = 0.008 for individuals; *p* = 0.041 for flocks). As ALB concentration gradually decreases in infectious and inflammatory diseases [60], the results indicate a chronic inflammatory reaction in turkeys with GL during early fattening. On the contrary, in the late fattening period, a higher globulin concentration was the cause of low A:G ratios in turkeys with GL (*p* ≤ 0.001 for individuals; *p* = 0.004 for flocks). A high globulin concentration can indicate an acute inflammatory process in which especially α-globulins increase rapidly [60]. To the knowledge of the authors, no reference values for the A:G ratio in turkeys have been published. However, A:G ratios overall were relatively low compared to published values for other bird species [22,58].

Higher GPX activities (relative to the PCV) were measured in individuals with GL and in hens from flocks with the occurrence of GL versus those without in the early fattening stage (*p* = 0.004 for individuals; *p* = 0.028 for flocks; Table 4 and Table 5). In the late fattening period, five turkey hens showed GPX activities that were distinctly higher than the reference values; all of these five turkeys had GL. The role of GPX in the antioxidant response in erythrocytes is mainly to catalyze the conversion of hydrogen peroxide to water and the reduction of other hydroperoxides [62]. GPX activity depends on a sufficient feed Se concentration, with a plateau of GPX activity being reached when the demand is met [34,40,62]. Ognik and Krauze [17] and Wang et al. [63] found an overall decrease in GPX activity during oxidative stress. However, the authors of [17] postulated that there is a rise in antioxidant enzyme mobility during oxidative stress, so GPX activity may increase in the initial period of a pathological state. Once antioxidants like glutathione or required trace elements like Se are depleted, the activity decreases [17].

Summing up the inflammation parameters of the turkeys examined at fattening days 120 to 127, it is assumed that individual Bronze turkey hens with GL undergo an acute inflammation process (high numbers of heterophils, high globulin concentration, and high GPX activity in some individuals). On the contrary, at days 70 to 75 of fattening, a more chronic inflammation process seems to take place in flocks affected by GL (high lymphocyte counts in individuals, high leucocyte numbers in affected flocks, and low ALB on both the flock and the individual level). These results complement the findings by Cuta et al. [12] who found reduced body weights and a higher prevalence of inflammatory reaction within the livers of turkey hens with GL compared to turkeys without liver discoloration during both stages of the fattening period. A correlation was shown between GL at 70 to 75 days of fattening and the detection of hemorrhagic enteritis virus as an immunosuppressive agent as well as GL at 120 to 127 days of fattening and bone or joint lesions or both, suggesting 2 different pathogeneses of the disease [12].

Regarding liver enzymes, GLDH activity is considered the most specific parameter for damaged hepatocytes in birds [21,23]. Given its mitochondrial localization, an increase in GLDH blood concentration is associated with severe liver damage [23,64]. GLDH activity was higher in Bronze turkey hens with GL as opposed to those without GL in the early fattening stage (*p* = 0.026; Table 4). Twelve hens showed GLDH concentrations above 10 U/L which is considered a clear sign of liver cell damage; 6 of them had GL [23]. Consistent with the fact that GLDH only increases due to severe liver cell damage, no significant differences between affected and unaffected flocks were seen (Table 5). GGT activity is increased in hepatobiliary obstruction and in some species during hepatic insult [23,64]. Only seven samples showed GGT activity above the detection limit, out of which three turkeys had GL. Each of these seven GGT values was within the physiological activity range [17]. Bile acid concentrations, which can rise in cases of reduced hepatic uptake function or reduced secretion into the intestine, were higher in both individuals and flocks with GL in the early fattening stage, but not on the later examination date, although both differences were not statistically significant [65]. No reference values for BA have been published specifically for turkeys, but values were within the physiological ranges reported for other bird species [22]. These findings possibly indicate an affected liver function and a difference in disease onset and time course for the two examination dates. Decreased ALB can be a sign of hepatic insufficiency but is rarely documented [23]. Higher AP (*p* = 0.022), AST (*p* = 0.028), CK (*p* = 0.008; *p* = 0.001), or LDH (*p* = 0.001) values were determined for individuals or flocks or both without GL at variable examination dates (Table 4 and Table 5). A variety of avian tissues contain AST, including the liver, skeletal muscle, heart muscle, kidney, and brain. Elevated plasma AST can be a sign of severe hepatic insult but seems to occur regularly in avian patients with muscle damage [21,23]. A concomitant increase in CK is a strong indicator of muscular insult [65]. AP in birds is a marker of increased osteoblastic activity and has low hepatobiliary activity [21,65]. LDH is widely distributed in a variety of avian tissues [21,64]. Connections between observed significant differences in AP, AST, CK, and LDH levels and liver function or the occurrence of GL thus cannot be derived.

Cholesterol is used as a marker for increased fat metabolism, hepatic fibrosis, extrahepatic biliary obstruction, or bile duct hyperplasia in birds [23,57]. Decreased plasma cholesterol may result from hepatic insufficiency or decreased dietary fat intake [66]. Cholesterol was lower in individuals with, as compared to those without, GL at the early fattening stage (*p* = 0.033; Table 4). LDL is a lipoprotein complex that delivers cholesterol to cells; plasma levels are dependent on feed intake and have not yet been studied extensively in turkeys [24,65,67]. Cholesterol (*p* ≤ 0.001) and LDL (*p* = 0.049) plasma levels were lower in the turkeys from flocks with than from flocks without the occurrence of GL (Table 5); however, all values were within described reference ranges for turkeys (cholesterol) or other bird species (LDL), respectively [15,19,67]. A correlation with the occurrence of GL is not assumed.

In the late fattening period, Bronze turkey hens with GL showed higher levels of Ca (*p* = 0.002) and a higher Ca:Pi ratio (*p* ≤ 0.001; Table 4), with values being within reference ranges [13,18]. The measured total Ca (other than ionized Ca) is bound to plasma proteins and therefore strongly influenced by the protein concentration in the blood. A significant correlation between Ca and ALB concentrations, as well as TP concentration has been documented in different avian species [59]. ALB and TP concentrations in the examined blood samples were also high in samples with high Ca concentrations, so a relationship between Ca levels and GL cannot be deducted.

### 3.3. Vitamin E and Selenium Liver Concentration

The analyzed vitamin E liver concentrations were within the wide range of published reference values [38,39]. The Se liver concentration was higher than described in the literature in many liver samples. However, the given reference values for hepatic Se concentrations were measured in turkeys receiving a diet with a maximum concentration of 0.38 Se mg/kg OS in a so-called pre-starter feed and 0.39 mg/kg OS in the starter feed [37,40,63], whereas the Se feed concentration in this study was higher in many samples (Table S6). There were no statistically significant differences between turkeys or flocks with and without GL regarding Se concentrations relative to DM. Relative to fresh matter (FM), turkeys without GL had a higher Se liver concentration than those with GL in the late fattening stage (*p* = 0.017; Table 6 and Table 7). Fischer et al. [40] found a Se concentration of 0.3 mg/kg OS to be necessary for sufficient GPX function in the liver and plasma of male (heavy) B.U.T. 6 turkeys. No similar studies have been performed for lighter or female turkeys. This minimum concentration was provided in all examined feed samples and all supply phases (calculated at 88% DM; Tables S6 and S11). Therefore, a sufficient Se supply and thus a sufficient GPX function can be assumed for all examined turkeys.

The vitamin E liver concentration was, although not statistically significant, lower in individuals with versus those without GL on both examination dates (Table 6). The vitamin E liver concentration was also lower in turkeys from flocks with the occurrence of GL on both examination dates. This difference was statistically significant in the late fattening stage (*p* = 0.049; Table 7). These results indicate either that flocks with the occurrence of GL are under oxidative stress and have a higher need for vitamin E, or that turkeys in flocks with a lower vitamin E supply are more likely to develop GL. However, there was no significant difference in feed vitamin E concentrations between flocks with and without GL on both examination dates, and a sufficient vitamin E supply can be assumed for all examined Bronze turkeys. Therefore, a higher consumption during oxidative stress seems likely. No direct influence of vitamin E or Se feed or liver concentration on the development of GL was determined, and it was not expected, assuming an adequate supply in all examined flocks.

### 3.4. Feed Analyses

The results of the feed sample analysis were generally quite homogeneous (Tables S1–S7). Variations in feed ingredient concentrations were noticeable in a few samples (one sample with exceptionally high levels of bulk and trace elements and vitamins E and D_3_, one sample with remarkably low levels of Ca, trace elements, and vitamins, and all five samples from one farm during one trial with strikingly high levels of CFAT, AMEn, and SU). No toxic levels were reached, and no negative effects could be identified (no abnormally high or low Ca or Pi blood levels or vitamin E or Se levels in the livers of turkeys from the corresponding flock). A general feature of feed from all flocks was the energy level dropping progressively (in feed samples as well as calculated supply phases) below the recommended level in the late phases of the fattening period [31,55], starting mainly in phase 2 (Tables S2 and S8). Furthermore, the concentration of (semi-)essential amino acids was generally lower than that recommended in all phases (for Met and Lys) or the late phases (Thr, Met + Cys; Tables S2, S3, S9 and S10). However, both energy and amino acid levels in the feed samples were in accordance with the manufacturers’ declarations. This reflects the difficulty of producing turkey diets according to recommendations under organic farming conditions in the European Union. Supplementation of synthetically manufactured amino acids is not allowed, and feed ingredients need to be grown ecologically and to a certain, varying extent (according to the requirements of different organic farmers’ associations) from own production or regional cooperating companies, which makes it hard to develop an adequate diet [52,68,69]. A derogation allowing the use of a maximum of 5% conventionally grown protein sources over a one year period has been extended several times and is currently valid up to and including 2025 [43,69,70,71,72]. Another general feature of most of the samples was the ASH concentration which was generally well above the manufacturers’ declarations (more than double in some samples). This originated from trace element concentrations that were constantly above declared levels and regularly exceeded legal limits. Out of 52 samples, the legal limit for Zn (120 mg/kg OS at 88% DM) and Se (0.5 mg/kg OS at 88% DM) was exceeded in 45 samples (86.54%) and 43 samples (82.69%), respectively [33,73]. Three samples (5.77%) exceeded the legal limit for Mn concentration (150 mg/kg OS at 88% DM), whereas one sample (1.92%) was above the legal limit for vitamin D₃ concentration (5000 IU/kg OS at 88% DM) [74,75].

Some of the results of the feed analysis in this study differ significantly from the results of the feed analysis of organically reared turkeys by Krautwald-Junghanns et al. [6]. In this previous study, the energy and nutrient contents were more heterogeneous between feed samples of the same phase, and deficiencies in bulk and trace element levels were found in many samples. Energy levels in the early phases were higher compared to the feed analyzed in this study and also higher than recommended, which can cause a reduction in total feed intake and contradicts organic feeding strategies [52]. Energy levels increased only slightly over the fattening period and therefore remained below recommended levels in the late feeding phases [6]. Amino acid concentrations (Lys, Met, Cys, Thr, Val, His) were often lower than recommended which is similar to the current study. However, average amino acid levels were also lower than in the current study in the early feeding phases (phases 0 to 2), and higher in the late fattening period (phases 4 and 5) [6].

To find a possible relation between the prevalence of GL within a turkey flock and the analyzed feed energy and nutrient content, the concentration offered within each feeding phase was checked for correlation with GL prevalence on both examination dates (for the early fattening stage only up to phase 3, as the examination took place within this phase). A negative correlation between GL prevalence and the supply of several amino acids was striking for both examination dates (*p* ≤ 0.05 for all correlations; Table 8). 

As mentioned before, the amino acid supply was lower than recommended, especially in the late fattening period. Of the amino acids that are regarded as most important for the avian immune system, Arg and Lys showed a negative correlation with GL prevalence (*p* ≤ 0.05), but not Met and Thr [53]. In the predecessor study, where a distinctly higher prevalence of GL was found at slaughter compared to each examination time point in the current study, deficient levels of Met, Cys, and Lys were documented in many feed samples [6,32]. Tykałowski et al. [30] found that Met feed content moderately affected the immune response of HEV-infected female Hybrid Converter turkeys. Boyeh et al. [76] showed that higher Met and Lys feed concentrations led to higher blood lymphocyte counts and lower heterophil counts in broilers. In order to look for similar direct influences of energy and nutrient feed contents on the immune system, nutrient and energy concentrations in each feeding phase and blood values and vitamin E and Se liver concentrations were examined for correlations on both examination dates. As many single positive and negative correlations were calculated, only those determined to be significant for at least three feeding phases are shown in Table 9. Only a few correlations were found for at least three phases, and no similar relationships have been described in the literature. A positive correlation was found between feed vitamin D₃ and vitamin E liver concentrations (*p* ≤ 0.05). It is known that vitamin D₃ is involved in the immune response and can increase disease resistance [26,77]. Higher feed concentrations might lead to a lower vitamin E demand for antioxidant protection. No explanation can be given for the other observed correlations and their meaning is questionable. 

Overall, the homogeneity of the results of the feed analyses leaves the calculation of correlations difficult. For example, the amino acid supply was deficient for all examined flocks in some phases, so there is no control group with a sufficient supply that could be compared. The evaluation of energy and nutrient supply in each phase was carried out with reservation because only the time periods in which certain feeds were offered could be calculated, but not the actual feed intake, as this had not been documented in sufficient detail on the farms. Additionally, only one batch of each phase feed was analyzed per trial and flock, while several batches were fed during each phase, especially towards the end of fattening. Furthermore, grains, dietary supplements which were additionally fed in most of the flocks, and—in one flock—vegetables could not be considered when calculating the turkeys’ supply within the different phases, because the fed amounts were not documented in detail. The examined grains differed greatly from the complete feed samples (lower levels of ASH, CP, amino acids, bulk and trace elements, and vitamins; Tables S1–S6). Moreover, the organically reared turkeys in this study (with some exceptions due to avian influenza restrictions) had access to a pasture and therefore to plants and insects. The change to a different turkey genetic base on the farms compared to previous studies could possibly influence susceptibility to feed deficiencies. The sample size for statistical analyses of the feed samples in each phase was comparatively small (*n* = 10 for the early fattening stage and *n* = 8 for the late fattening stage due to two canceled examinations).

However, the fact that GL prevalence was significantly higher in the predecessor study on organically reared turkeys and the results of the feed analyses showed more deficiencies back then suggests that a better diet composition may contribute to reducing GL prevalence [6]. In order to investigate the direct influences of the feeding regime on the occurrence of GL, studies under experimental settings are needed. The correlation between GL prevalence and low amino acid supply as well as the appropriate supply of bulk and trace elements in this study compared to earlier studies with a higher prevalence of GL encourage further research. It is well known that an insufficient diet has a significant impact on the avian immune response and therefore on the ability of poultry to resist infectious agents [27,28]. 

## 4. Conclusions

The findings of the performed blood and liver analyses in this study give insight into the formal pathogenesis of green liver discoloration, suggesting a flock-specific subacute etiology at the age of 70 to 75 days and an individual, more acute etiology at the age of 120 to 127 days of fattening. This complements the findings concerning the causal pathogenesis of GL by Cuta et al. [12] who presented a correlation of GL with the immunosuppressive hemorrhagic enteritis virus during the early fattening stage and joint or bone lesions or both in the late fattening stage. These results support the previously made assumption that a weakened immune system plays a bigger role in the development of GL than a single pathogen [11]. 

An appropriate diet meeting turkeys’ nutritional requirements is likely to reduce the occurrence of GL by allowing the proper function of turkey immune systems. This study presents deficiencies in commercial turkey feed (energy and amino acid concentration below recommendations, trace element levels exceeding legal limits) and hints at a correlation with the development of GL and therefore suggests approaches for improvement. However, due to the limitations of the study, no direct influence of certain nutrients on the pathogenesis of GL can be proven with certainty, and further research under experimental conditions is recommended.

## Figures and Tables

**Table 1:** Green liver prevalence in examined hen flock samples from five different Bronze turkey farms during two consecutive fattening trials.

Trial	Examination Time Point	Farm 1	Farm 2	Farm 3	Farm 4	Farm 5
1	70 to 75 Days of Fattening	*n*= 18 *	*n* = 20	*n* = 20	*n* = 19 *	*n* = 19 *
		0.0%	0.0%	0.0%	68.4%	0.0%
	120 to 127 Days of Fattening	*n* = 20	*n* = 20	*n* = 20	N.a.	N.a.
		0.0%	10.0%	10.0%		
2	70 to 75 Days of Fattening	*n* = 20	*n* = 11 *	*n* = 20	*n* = 20	*n* = 17 *
		5.0%	9.1%	0.0%	5.0%	0.0%
	120 to 127 Days of Fattening	*n* = 20	*n* = 20	*n* = 20	*n* = 20	*n* = 19 *
		10.0%	0.0%	0.0%	20.0%	26.3%

* If *n* < 20, male turkeys were falsely included in the sample and were not further included in the study; N.a.: Not applicable (collection of samples was not possible due to avian influenza control measures and the SARS-CoV-2 pandemic).

**Table 2:** Comparison of hematological values of female Bronze turkey individuals with and without green liver discoloration in the early fattening stage (70 to 75 days of fattening) and the late fattening stage (120 to 127 days of fattening), respectively (comparison of mean ($\bar{x}$) or *median* ($\tilde{x}$) ± standard error of mean or *median* (SE) within the same age).

Hematological Value	70 to 75 Days of Fattening			120 to 127 Days of Fattening		
	Green Liver	No Green Liver		Green Liver	No Green Liver	
	*n* = 9/8 ^1^	*n* = 58/56 ^1^		*n* = 15/14 ^1^	*n* = 48/46 ^1^	
	$\bar{x}$ $/\tilde{x}\pm \mathrm{SE}$	$\bar{x}$ $/\tilde{x}\pm \mathrm{SE}$	*p*-Value	$\bar{x}$ $/\tilde{x}\pm \mathrm{SE}$	$\bar{x}$ $/\tilde{x}\pm \mathrm{SE}$	*p*-Value
PCV (L/L)	0.34 ± 0.01	0.35 ± 0.01	0.309	0.34 ± 0.01	0.38 ± 0.01	<0.001
Hb (mmol/L)	*5.81 ± 0.22*	*5.44 ± 0.10*	*0.164*	5.39 ± 0.23	5.83 ± 0.09	0.033
WBC (×10^9^/L)	*29.85 ± 5.54*	*24.45 ± 1.34*	*0.011*	*33.15 ± 7.18*	*27.15 ± 1.04*	*0.007*
Heterophils (×10^9^/L)	*14.28 ± 4.56*	*10.59 ± 0.87*	*0.063*	*17.24 ± 8.36*	*11.51 ± 0.89*	*0.025*
Lymphocytes (×10^9^/L)	14.10 ± 1.71	10.65 ± 0.62	0.048	13.28 ± 1.14	12.78 ± 0.53	0.667
Eosinophils (×10^9^/L)	*0.00 ± 0.14*	*0.00 ± 0.05*	*0.505*	*0.13 ± 0.14*	*0.00 ± 0.08*	*0.495*
Basophils (×10^9^/L)	*2.08 ± 0.68*	*1.26 ± 0.12*	*0.104*	*1.04 ± 0.20*	*1.20 ± 0.11*	*0.722*
Monocytes (×10^9^/L)	*0.78 ± 0.35*	*0.47 ± 0.12*	*0.351*	*0.92 ± 0.39*	*0.29 ± 0.08*	*0.088*
H:L ratio	*1.22 ± 0.50*	*1.20 ± 0.09*	*0.883*	*1.27 ± 0.69*	*0.91 ± 0.09*	*0.030*

^1^ Number of cases lower for PCV and Hb; Hb: hemoglobin; H:L: heterophile:lymphocyte; PCV: packed cell volume; WBC: white blood cells.

**Table 3:** Comparison of hematological values of Bronze turkey hens from flocks with and without the occurrence of green liver discoloration in the early fattening stage (70 to 75 days of fattening) and the late fattening stage (120 to 127 days of fattening), respectively (comparison of mean ($\bar{x}$) or *median* ($\tilde{x}$) ± standard error of mean or *median* (SE) within the same age).

Hematological Value	70 to 75 Days of Fattening			120 to 127 Days of Fattening		
	Green Liver(s)	No Green Liver		Green Liver(s)	No Green Liver	
	*n* = 31/30 ^1^	*n* = 36/34 ^1^		*n* = 45/40 ^1^	*n* = 18	
	$\bar{x}$ $/\tilde{x}\pm \mathrm{SE}$	$\bar{x}$ $/\tilde{x}\pm \mathrm{SE}$	*p*-Value	$\bar{x}$ $/\tilde{x}\pm \mathrm{SE}$	$\bar{x}$ $/\tilde{x}\pm \mathrm{SE}$	*p*-Value
PCV (L/L)	0.35 ± 0.01	0.35 ± 0.01	0.646	*0.38 ± 0.004*	*0.38 ± 0.01*	*0.993*
Hb (mmol/L)	*5.54 ± 0.14*	*5.47 ± 0.15*	*0.767*	5.78 ± 0.12	5.60 ± 0.10	0.370
WBC (×10^9^/L)	*27.60 ± 1.60*	*21.53 ± 1.98*	*0.003*	*27.90 ± 1.72*	*27.98 ± 1.86*	*0.939*
Heterophils (×10^9^/L)	*14.28 ± 1.47*	*9.59 ± 0.94*	*0.010*	*12.75 ± 1.22*	*13.32 ± 2.55*	*0.819*
Lymphocytes (×10^9^/L)	12.10 ± 0.95	10.27 ± 0.73	0.127	12.77 ± 0.54	13.23 ± 1.02	0.668
Eosinophils (×10^9^/L)	*0.00 ± 0.07*	*0.00 ± 0.06*	*0.680*	*0.13 ± 0.10*	*0.00 ± 0.04*	*0.105*
Basophils (×10^9^/L)	*1.54 ± 0.23*	*0.97 ± 0.13*	*<0.001*	*1.23 ± 0.14*	*0.94 ± 0.27*	*0.041*
Monocytes (×10^9^/L)	*0.82 ± 0.19*	*0.41 ± 0.08*	*0.034*	*0.41 ± 0.17*	*0.33 ± 0.12*	*0.866*
H:L ratio	*1.31 ± 0.14*	*1.10 ± 0.12*	*0.285*	*1.04 ± 0.08*	*0.87 ± 0.23*	*0.784*

^1^ Number of cases lower for PCV and Hb; Hb: hemoglobin; H:L: heterophile:lymphocyte; PCV: packed cell volume; WBC: white blood cells.

**Table 4:** Comparison of clinical chemistry values of female Bronze turkey individuals with and without green liver discoloration in the early fattening stage (age 70 to 75 days of fattening) and the late fattening stage (age 120 to 127 days of fattening), respectively (comparison of mean ($\bar{x}$) or *median* ($\tilde{x}$) ± standard error of mean or *median* (SE) within the same age).

Clinical Chemistry Value	70 to 75 Days of Fattening			120 to 127 Days of Fattening		
	Green Liver	No Green Liver		Green Liver	No Green Liver	
	*n* = 9/8 ^1^	*n* = 58/56 ^1^		*n* = 15/14 ^1^	*n* = 48/47 ^2^/46 ^1^	
	$\bar{x}$ $/\tilde{x}\pm \mathrm{SE}$	$\bar{x}$ $/\tilde{x}\pm \mathrm{SE}$	*p*-Value	$\bar{x}$ $/\tilde{x}\pm \mathrm{SE}$	$\bar{x}$ $/\tilde{x}\pm \mathrm{SE}$	*p*-Value
ALB (g/L)	*9.8 ± 0.8*	*11.2 ± 0.2*	*0.008*	13.0 ± 0.4	12.3 ± 0.2	0.027
A:G ratio	*0.35 ± 0.01*	*0.38 ± 0.01*	0.003	0.37 ± 0.02	0.47 ± 0.01	<0.001
AP (U/L)	*1234 ± 110*	*1426 ± 36*	*0.022*	*762 ± 99*	*811 ± 27*	*0.080*
AST (U/L)	*326.3 ± 48.3*	*335.8 ± 8.1*	*0.699*	*474.3 ± 43.5*	*590.9 ± 31.6*	*0.066*
BA (µmol/L)	*16.6 ± 3.8*	*12.2 ± 1.2*	*0.278*	*9.8 ± 3.8*	*10.7 ± 1.9*	*0.729*
Ca (mmol/L)	*2.90 ± 0.15*	*2.60 ± 0.08*	*0.132*	*3.11 ± 0.25*	*2.62 ± 0.05*	*0.002*
Ca:Pi ratio	1.25 ± 0.05	1.14 ± 0.04	0.061	*1.57 ± 0.12*	*1.30 ± 0.04*	*<0.001*
CHOL (mmol/L)	1.88 ± 0.25	2.53 ± 0.06	0.033	2.84 ± 0.17	3.20 ± 0.06	0.067
CK (U/L)	*3232 ± 1599*	*4107 ± 447*	*0.927*	*10,450 ± 2148*	*200,647 ± 2313*	*0.008*
GLDH (U/L)	*6.4 ± 7.2*	*4.0 ± 0.3*	*0.026*	*3.6 ± 1.5*	*3.7 ± 0.4*	*0.735*
Globulin (g/L)	*29.7 ± 2.12*	*29.0 ± 0.77*	*0.847*	*35.4 ± 2.84*	*26.1 ± 0.49*	*<0.001*
GPX (U/mL PCV)	*55.08 ± 3.45*	*42.11 ± 2.72*	*0.004*	*52.76 ± 18.01*	*49.91 ± 1.44*	*0.151*
GPX (U/g Hb)	*196.16 ± 27.12*	*137.13 ± 10.20*	*0.051*	*193.95 ± 80.20*	*161.80 ± 10.35*	*0.156*
LDH (U/L)	*787 ± 74*	*622 ± 37*	*0.051*	*736 ± 103*	*745 ± 43*	*0.117*
LDL (mmol/L)	0.66 ± 0.10	0.83 ± 0.03	0.143	*0.86 ± 0.13*	*0.86 ± 0.04*	*0.916*
Mg (mmol/L)	0.89 ± 0.04	0.89 ± 0.02	0.993	*0.92 ± 0.03*	*0.91 ± 0.01*	*0.241*
Pi (mmol/L)	*2.28 ± 0.11*	*2.33 ± 0.04*	*0.587*	*1.90 ± 0.07*	*1.97 ± 0.03*	*0.362*
TG (mmol/L)	*0.59 ± 0.11*	*0.49 ± 0.02*	*0.550*	*0.40 ± 0.11*	*0.44 ± 0.01*	*0.686*
TP (g/L)	*40.0 ± 2.9*	*39.9 ± 0.9*	*0.435*	*47.5 ± 3.4*	*38.6 ± 0.6*	*<0.001*
UA (µmol/L)	*141 ± 51*	*142 ± 8*	0.526	190 ± 16	217 ± 11	0.200

^1^ Number of cases lower for GPX, ^2^ number of cases lower for Ca, Ca:Pi ratio, and Mg; ALB: Albumin; A:G: albumin:globulin; AP: alkaline phosphatase; AST: aspartate aminotransferase; BA: bile acids; Ca: calcium; Chol: cholesterol; CK: creatin kinase; GLDH: glutamate dehydrogenase; GPX: glutathione peroxidase; LDH: lactate dehydrogenase; LDL: low density lipoprotein; Mg: magnesium; Pi: inorganic phosphate; TP: total protein; TG: triglycerides; UA: uric acid.

**Table 5:** Comparison of clinical chemistry values of Bronze turkey hens from flocks with and without the occurrence of green liver discoloration in the early fattening stage (70 to 75 days of fattening) and the late fattening stage (120 to 127 days of fattening), respectively (comparison of mean ($\bar{x}$) or *median* ($\tilde{x}$) ± standard error of mean or *median* (SE) within the same age).

Clinical Chemistry Value	70 to 75 Days of Fattening			120 to 127 Days of Fattening		
	Green Liver(s)	No Green Liver		Green Liver(s)	No Green Liver	
	*n* = 31/30 ^1^	*n* = 36/34 ^1^		*n* = 45/44 ^2^/40 ^1^	*n* = 18	
	$\bar{x}$ $/\tilde{x}\pm \mathrm{SE}$	$\bar{x}$ $/\tilde{x}\pm \mathrm{SE}$	*p*-Value	$\bar{x}$ $/\tilde{x}\pm \mathrm{SE}$	$\bar{x}$ $/\tilde{x}\pm \mathrm{SE}$	*p*-Value
ALB (g/L)	*10.6 ± 0.3*	*11.2 ± 0.2*	*0.041*	12.6 ± 0.2	12.1 ± 0.2	0.113
A:G ratio	0.37± 0.01	0.40 ± 0.01	0.039	*0.45 ± 0.01*	*0.48 ± 0.01*	*0.001*
AP (U/L)	*1392 ± 43*	*1433 ± 50*	*0.439*	815 ± 29	820 ± 28	0.919
AST (U/L)	*326.3 ± 17.9*	*336.8 ± 7.4*	*0.365*	*513.7 ± 38.7*	*668.1 ± 45.3*	*0.018*
BA (µmol/L)	*15.0 ± 1.6*	*10.6 ± 1.6*	*0.172*	*9.8 ± 2.0*	*12.1 ± 3.0*	*0.223*
Ca (mmol/L)	2.69 ± 0.07	2.59 ± 0.06	0.336	*2.67 ± 0.05*	*2.72 ± 0.09*	*0.907*
Ca:Pi ratio	1.18 ± 0.03	1.13 ± 0.03	0.182	*1.39 ± 0.03*	*1.27 ± 0.04*	*0.031*
CHOL (mmol/L)	2.20 ± 0.09	2.66 ± 0.08	<0.001	3.05 ± 0.08	3.26 ± 0.12	0.147
CK (U/L)	*3533 ± 403*	*4766 ± 619*	*0.291*	*12,879 ± 1517*	*24,711 ± 2214*	*0.001*
GLDH (U/L)	*3.6 ± 0.5*	*4.4 ± 0.5*	*0.352*	*3.5 ± 0.3*	*4.4 ± 0.3*	*0.185*
Globulin (g/L)	*29.1 ± 0.6*	*29.0 ± 1.3*	*0.501*	*27.7 ± 0.8*	*25.8 ± 0.9*	*0.004*
GPX (U/mL PCV)	46.49 ± 2.49	39.53 ± 1.90	0.028	*49.46 ± 1.44*	*52.02 ± 4.62*	*0.430*
GPX (U/g Hb)	*129.02 ± 14.06*	*149.01 ± 10.18*	*0.226*	*161.03 ± 12.18*	*207.86 ± 20.24*	*0.060*
LDH (U/L)	*585 ± 556*	*664 ± 34*	*0.282*	*674 ± 54*	*909 ± 91*	*0.001*
LDL (mmol/L)	0.75 ± 0.04	0.86 ± 0.04	0.049	*0.85 ± 0.04*	*0.92 ± 0.08*	*0.615*
Mg (mmol/L)	0.89 ± 0.01	0.88 ± 0.01	0.606	*0.92 ± 0.01*	*0.92 ± 0.02*	*0.658*
Pi (mmol/L)	*2.32 ± 0.05*	*2.31 ± 0.05*	*0.758*	*1.95 ± 0.03*	*2.02 ± 0.11*	*0.088*
TG (mmol/L)	*0.52 ± 0.03*	*0.48 ± 0.03*	*0.225*	*0.45 ± 0.03*	*0.43 ± 0.03*	*0.270*
TP (g/L)	*39.9 ± 0.6*	*40.1 ± 1.7*	*0.396*	*40.3 ± 0.9*	*37.8 ± 1.2*	*0.005*
UA (µmol/L)	*136 ± 11*	*145 ± 8*	*0.619*	*198 ± 11*	*221 ± 24*	*0.605*

^1^ Number of cases lower for GPX, ^2^ number of cases lower for Ca, Ca:Pi ratio and Mg; ALB: Albumin; A:G: albumin:globulin; AP: alkaline phosphatase; AST: aspartate aminotransferase; BA: bile acids; Ca: calcium; Chol: cholesterol; CK: creatin kinase; GLDH: glutamate dehydrogenase; GPX: glutathione peroxidase; LDH: lactate dehydrogenase; LDL: low density lipoprotein; Mg: magnesium; Pi: inorganic phosphate; TP: total protein; TG: triglycerides; UA: uric acid.

**Table 6:** Comparison of vitamin E and selenium liver concentrations of female Bronze turkey individuals with and without green liver discoloration in the early fattening stage (70 to 75 days of fattening) and the late fattening stage (120 to 127 days of fattening), respectively (comparison of mean ($\bar{x}$) or *median* ($\tilde{x}$) ± standard error of mean or *median* (SE) within the same age).

Liver Concentration	70 to 75 Days of Fattening			120 to 127 Days of Fattening		
	Green Liver	No Green Liver		Green Liver	No Green Liver	
	*n* = 9	*n* = 58		*n* = 15	*n* = 48	
	$\bar{x}$ $/\tilde{x}\pm \mathrm{SE}$	$\bar{x}$ $/\tilde{x}\pm \mathrm{SE}$	*p*-Value	$\bar{x}$ $/\tilde{x}\pm \mathrm{SE}$	$\bar{x}$ $/\tilde{x}\pm \mathrm{SE}$	*p*-Value
Vitamin E (µg/g FM)	2.08 ± 0.13	2.76 ± 1.27	0.074	4.12 ± 0.42	4.62 ± 0.22	0.284
Selenium (µg/g DM)	*1.60 ± 0.12*	*1.49 ± 0.04*	*0.116*	1.61 ± 0.09	1.74 ± 0.03	0.117
Selenium (µg/g FM)	*0.45 ± 0.03*	*0.41 ± 0.01*	*0.289*	0.45 ± 0.03	0.50 ± 0.01	0.017

DM: dry matter; FM: fresh matter.

**Table 7:** Comparison of vitamin E and selenium liver concentrations of Bronze turkey hens from flocks with and without the occurrence of green liver discoloration in the early fattening stage (70 to 75 days of fattening) and the late fattening stage (120 to 127 days of fattening), respectively (comparison of mean ($\bar{x}$) or *median* ($\tilde{x}$*)* ± standard error of mean or *median* (SE) within the same age).

Liver Concentration	70 to 75 Days of Fattening			120 to 127 Days of Fattening		
	Green Liver(s)	No Green Liver		Green Liver(s)	No Green Liver	
	*n* = 31	*n* = 36		*n* = 45	*n* = 18	
	$\bar{x}$ $/\tilde{x}\pm \mathrm{SE}$	$\bar{x}$ $/\tilde{x}\pm \mathrm{SE}$	*p*-Value	$\bar{x}$ $/\tilde{x}\pm \mathrm{SE}$	$\bar{x}$ $/\tilde{x}\pm \mathrm{SE}$	*p*-Value
Vitamin E (µg/g FM)	2.62 ± 0.21	2.72 ± 0.17	0.694	4.26 ± 0.23	5.11 ± 0.34	0.049
Selenium (µg/g DM)	*1.49 ± 0.05*	*1.50 ± 0.05*	*0.930*	1.75 ± 0.05	1.61 ± 0.06	0.175
Selenium (µg/g FM)	*0.41 ± 0.02*	*0.42 ± 0.01*	*0.757*	0.50 ± 0.01	0.48 ± 0.02	0.476

DM: dry matter; FM: fresh matter.

**Table 8:** Statistically significant correlations between energy and nutrient supply during recommendation phases 0 to 5 and green liver prevalence on both examination dates (Pearson’s correlation coefficient (r) given in case of a normal distribution, Spearman’s rank correlation coefficient (ρ) given if data did not follow a normal distribution, negative correlations marked with minus sign; *p* ≤ 0.05 and >0.01 for all correlations).

Nutrient	Phase 0	Phase 1	Phase 2	Phase 3	Phase 4	Phase 5
Early Fattening Stage (70 to 75 Days of Fattening), *n* = 10						
Pro	ρ = 0.679	ρ = 0.634	-	ρ = −0.754	n.a.	n.a.
Ala	-	-	-	ρ = −0.836	n.a.	n.a.
Arg	-	-	-	ρ = −0.637	n.a.	n.a.
Glu	-	-	-	ρ = −0.706	n.a.	n.a.
Gly	-	-	-	ρ = −0.761	n.a.	n.a.
Lys per AMEn	-	-	-	ρ = −0.670	n.a.	n.a.
Phe	-	-	-	ρ = −0.761	n.a.	n.a.
Late Fattening Stage (120 to 127 Days of Fattening), *n* = 8						
Arg	r = −0.775	-	-	-	-	-
Asp	r = −0.722	-	-	-	-	-
CP	-	r = 0.751	-	-	-	-
DM	-	-	r = −0.771	-	-	-
ASH	-	-	-	-	ρ = 0.726	-
Glu	-	-	-	-	r = −0.737	-
Pro	-	-	-	-	r = −0.804	-
Ser	-	-	-	-	ρ = −0.726	-
Starch	-	-	-	-	r = 0.843	-
SU	-	-	-	-	-	r = −0.902
Thr	-	-	-	-	-	r = −0.778

Ala: alanine; AMEn: apparent metabolizable energy corrected by nitrogen; Arg: arginine; ASH: crude ash; Asp: aspartic acid; CP: crude protein; DM: dry matter; Gly: glycine; Glu: glutamic acid; Lys: lysine; n.a.: not applicable; Phe: phenylalanine; Pro: proline; Ser: serine; SU: sugar; Thr: theonine.

**Table 9:** Significant correlations between energy and nutrient supply and averaged blood and liver values on both examination dates (only shown if correlation was significant over at least three out of six supply phases, therefore no correlation coefficients or further distinction between *p*-values are given (*p* ≤ 0.05 for all correlations); pos = positive correlation; neg = negative correlation)).

Blood or Liver Value	Nutrient in Diet						
	CF	Starch	Cys	Phe	Tyr	Vitamin D₃	Vitamin E
Early Fattening Stage (70 to 75 Days of Fattening), *n* = 10							
Magnesium	-	-	pos	-	-	-	-
Total protein	-	-	pos	-	-	-	-
Triglycerides	-	-	pos	-	-	-	-
Vitamin E liver	-	-	-	-	neg	pos	-
Late Fattening Stage (120 to 127 Days of Fattening), *n* = 8							
Alcaline phosphatase	pos	-	-	-	-	-	pos
Basophils	--	-	-	neg	neg	-	-
Ca:Pi ratio	-	pos	-	-	-	-	-

Ca: calcium; CF: crude fiber; Cys: cysteine; Phe: phenylalanine; Pi: inorganic phosphate; Tyr: tyrosine.

## Data Availability

The data presented in this review are available on request from the corresponding author.

## Supplementary Materials

The following supporting information can be downloaded at: https://www.mdpi.com/article/10.3390/ani13091558/s1, Table S1: Raw nutrient concentrations in feed samples (mean, minimum–maximum) referred to 88% DM; Table S2: Raw nutrient, energy, and amino acid concentrations in feed samples (mean, minimum–maximum) referred to 88% DM; Tables S3–S5: Amino acid concentrations in feed samples (mean, minimum–maximum) referred to 88% DM; Table S6: Bulk and trace element concentrations in feed samples (mean, minimum–maximum) referred to 88% DM; Table S7: Vitamin concentrations in feed samples (mean, minimum–maximum) referred to 88% DM; Table S8: Raw nutrient and energy concentrations in feed samples: Actual supply during recommendation periods of the Society of Nutrition Physiology [31] referred to 88% DM or MJ AMEn; A = mean (minimum–maximum), B = recommendation by [31,53], respectively; Tables S9 and S10: Amino acid concentrations in feed samples: Actual supply during recommendation periods of the Society of Nutrition Physiology [31] referred to 88% DM or MJ AMEn; A = mean (minimum–maximum), B = recommendation [31,53], respectively; Table S11: Bulk and trace element concentrations in feed samples: Actual supply during recommendation periods of the Society of Nutrition Physiology [31] referred to 88% DM; A = mean (minimum–maximum), B = recommendation [31]; Table S12: Vitamin concentrations in feed samples: Actual supply during recommendation periods of the Society of Nutrition Physiology [31] referred to 88% DM; A = mean (minimum–maximum), B = recommendation [31].
