# Supplementary material for: Investigation of the Formal Pathogenesis of Green Liver Discoloration in Organically Reared Female Bronze Turkeys (Melleagris gallovapo)
Source: Animals (Basel). 2023 May 6;13(9):1558. doi: 10.3390/ani13091558 (PMC10177269; doi:10.3390/ani13091558)
Supplement: Supplementary file 1 [file animals-13-01558-s001.zip › animals-2331021-supplementary.pdf]

Table S1. Raw nutrient concentrations in feed samples (mean, minimum - maximum) referred to 88% DM.

Feed sample	N	ASH (g/kg)	CFAT (g/kg)	CP (g/kg)	CF (g/kg)	NFE (g/kg)	Starch (g/kg)
Starter feed	11	76.44 (69.73 - 109.41)	57.54 (48.73 - 75.8)	281.42 (255.73 - 301.58)	37.83 (25.31 - 43.22)	426.86 (393.86 - 460.91)	273.61 (247.35 - 318.94)
Phase 1	11	71.35 (63.29 - 83.66)	53.17 (44.48 - 69.72)	268.13 (230.45 - 288.84)	38.44 (29.01 - 61.26)	448.94 (438.05 - 487.37)	296.92 (284.28 - 340.87)
Phase 2	10	65.76 (61.50 - 73.07)	49.11 (44.05 - 58.34)	237.96 (207.40 - 252.26)	40.66 (36.34 - 44.53)	486.57 (472.95 - 504.93)	346.82 (327.94 - 365.37)
Phase 3	10	59.18 (56.09 - 62.33)	43.73 (40.11 - 48.59)	176.63 (158.56 - 187.09)	40.28 (36.08 - 46.42)	560.07 (544.43 - 581.71)	417.67 (403.87 - 440)
Final fattening feed	10	57.49 (47.83 - 64.75)	42.00 (34.59 - 60.95)	155.18 (145.85 - 166.78)	38.32 (34.73 - 41.78)	587.14 (567.77 - 601.02)	440.12 (411.66 - 461.05)
Oat	5	26.33 (24.09 - 28.41)	43.29 (34.73 - 60.84)	97.42 (82.77 - 113.55)	99.19 (87.22 - 118.39)	613.62 (602.78 - 620.25)	398.59 (351.21 - 427.65)
Wheat	1	15.35	21.02	92.20	25.62	725.51	607.20

ASH: crude ash; CF: crude fiber; CFAT: crude fat; CP: crude protein; DM: dry matter; NFE: nitrogen-free extracts.

Table S2. Raw nutrient, energy, and amino acid concentrations in feed samples (mean, minimum - maximum) referred to 88% DM.

Feed sample	N	SU (g/kg)	AMEn (kcal/kg)	AMEn (MJ/kg)	MET (g/kg)	MET + CYS (g/kg)
Starter feed	11	36.94 (30.14 - 46.83)	2,718.07 (2,612.97 – 2,799.27)	11.38 (10.94 - 11.72)	5.16 (4.15 - 6.15)	9.50 (8.35 - 10.59)
Phase 1	11	36.66 (31.34 - 44.73)	2,722.84 (2,665.52 – 2,782.55)	11.40 (11.16 - 11.65)	5.08 (3.27 - 5.80)	9.28 (6.77 - 10.96)
Phase 2	10	38.71 (34.13 - 42.45)	2,784.94 (2,718.07 – 2,870.93)	11.66 (11.38 - 12.02)	4.27 (3.63 - 4.91)	8.15 (6.92 - 9.44)
Phase 3	10	40.70 (37.78 - 43.71)	2,804.05 (2,761.06 – 2,856.60)	11.74 (11.56 - 11.96)	3.28 (2.71 - 4.26)	6.52 (5.51 - 7.6)
Final fattening feed	10	39.71 (37.22 - 42.68)	2,794.50 (2,694.18 – 2,873.32)	11.70 (11.28 - 12.03)	2.41 (1.92 - 3.27)	5.29 (4.44 - 6.26)
Oat	5	11.51 (10.95 - 12.20)	2,335.91 (2,073.18 – 2,548.49)	9.78 (8.68 - 10.67)	1.85 (1.57 - 2.27)	4.65 (4.03 - 5.00)
Wheat	1	28.84	3,035.73	12.71	1.84	3.92

AMEn: apparent metabolizable energy corrected by nitrogen; CYS: cysteine; DM: dry matter; MET: methionine; SU: sugar.

Table S3. Amino acid concentrations in feed samples (mean, minimum - maximum) referred to 88% DM.

Feed sample	N	LYS (g/kg)	THR (g/kg)	CYS (g/kg)	ASP (mg/kg)	SER (mg/kg)	GLU (mg/kg)
Starter feed	11	14.94 (13.52 - 16.06)	11.42 (9.85 – 15.60)	4.34 (3.96 – 4.96)	26.97 (23.73 – 28.95)	14.11 (12.45 – 16.35)	54.03 (50.98 – 58.86)
Phase 1	11	13.81 (11.86 - 15.02)	10.51 (8.14 – 11.55)	4.20 (3.50 – 5.17)	25.11 (22.36 -27.92)	13.58 (11.28 – 15.98)	52.81 (43.37 – 57.48)
Phase 2	10	12.29 (11.63 - 13.12)	9.12 (7.45 – 10.17)	3.88 (3.04 – 4.58)	22.65 (19.67 – 25.18)	11.88 (10.47 – 12.85)	48.69 (43.85 – 53.55)
Phase 3	10	8.97 (7.55 - 10.24)	6.66 (5.16 – 7.55)	3.24 (2.69 – 3.63)	16.89 (14.57 – 18.73)	8.68 (7.40 – 10.05)	38.98 (35.36 – 43.80)
Final fattening feed	10	7.55 (6.82 - 8.68)	5.48 (4.65 – 5.90)	2.88 (2.49 – 3.36)	14.13 (12.74 – 16.41)	7.40 (6.80 – 8.14)	36.53 (32.21 – 40.24)
Oat	5	4.16 (3.62 - 4.85)	3.58 (2.95 – 4.06)	2.81 (2.44 – 3.06)	8.07 (7.03 – 9.28)	4.70 (4.00 – 5.33)	21.18 (18.57 – 24.18)
Wheat	1	2.85	3.32	2.08	5.02	4.30	26.20

ASP: aspartic acid; GLU: glutamic acid; DM: dry matter; LYS: lysine; SER: serine; THR: threonine.

Table S4. Amino acid concentrations in feed samples (mean, minimum - maximum) referred to 88% DM.

Feed sample	N	GLY (mg/kg)	ALA (mg/kg)	VAL (mg/kg)	ISO (mg/kg)	LEU (mg/kg)	TYR (mg/kg)
Starter feed	11	13.16 (11.77 – 15.31)	15.27 (13.56 – 16.83)	14.16 (12.74 – 15.68)	12.09 (10.79 – 12.98)	25.54 (23.05 – 29.11)	10.29 (9.17 – 11.73)
Phase 1	11	12.61 (10.50 – 14.46)	14.88 (10.79 – 16.75)	13.41 (11.09 – 15.31)	11.34 (9.57 – 12.42)	24.76 (17.89 – 28.31)	9.79 (8.02 – 11.17)
Phase 2	10	10.91 (9.83 – 11.63)	12.23 (10.18 – 13.22)	11.81 (10.18 – 12.74)	10.00 (8.57 – 10.96)	20.74 (15.70 – 22.96)	9.16 (6.40 – 12.70)
Phase 3	10	8.31 (7.18 – 8.91)	8.04 (6.88 – 8.88)	8.81 (7.79 – 9.66)	7.40 (6.41 – 8.11)	13.67 (11.89 – 15.12)	5.44 (2.80 – 6.49)
Final fattening feed	10	7.05 (6.63 – 7.71)	6.77 (6.44 – 7.34)	7.39 (6.87 – 8.17)	6.20 (5.73 – 6.91)	11.39 (10.51 – 12.50)	4.90 (4.48 – 5.66)
Oat	5	5.08 (4.39 – 5.90)	4.77 (4.08 – 5.53)	5.11 (4.56 – 6.00)	3.57 (3.13 – 4.23)	7.13 (6.19 – 8.24)	3.16 (2.75 – 3.80)
Wheat	1	3.94	3.69	4.13	3.15	6.04	3.09

ALA: alanine; DM: dry matter; GLY: glycine; ISO: isoleucine; LEU: leucine; TYR: tyrosine; VAL: valine.

Table S5. Amino acid concentrations in feed samples (mean, minimum - maximum) referred to 88% DM.

Feed sample	N	PHE (mg/kg)	HIS (mg/kg)	ARG (mg/kg)	PRO (mg/kg)	OH-Proline (mg/kg)	TAU (mg/kg)
Starter feed	11	14.31 (12.84 – 15.87)	6.39 (5.82 – 6.80)	16.72 (14.97 – 17.46)	17.16 (15.61 – 21.98)	0 (0 – 0)	0 (0 – 0)
Phase 1	11	13.51 (11.28 – 15.21)	6.11 (5.53 – 6.57)	16.06 (14.55 – 17.42)	16.86 (12.74 – 20.41)	0 (0 – 0)	0 (0 – 0)
Phase 2	10	11.23 (8.79 – 13.32)	5.40 (4.89 – 5.90)	14.60 (13.71 – 15.92)	14.80 (12.50 – 16.95)	0 (0 – 0)	0 (0 – 0)
Phase 3	10	8.60 (7.51 – 9.43)	4.23 (3.84 – 4.70)	11.55 (10.60 – 12.39)	11.25 (9.61 – 12.07)	0 (0 – 0)	0 (0 – 0)
Final fattening feed	10	7.32 (6.73 – 8.20)	3.76 (3.55 – 4.10)	10.46 (9.80 – 11.92)	9.97 (8.97 – 11.63)	0 (0 – 0)	0 (0 – 0)
Oat	5	4.76 (4.17 – 5.59)	2.09 (1.81 – 2.51)	6.52 (5.61 – 7.87)	5.10 (4.41 – 5.78)	0 (0 – 0)	0 (0 – 0)
Wheat	1	4.08	2.12	4.64	8.18	0	0

ARG: arginine; DM: dry matter; HIS: histidine; PHE: phenylalanine; PRO: proline; TAU: taurine.

Table S6. Bulk and trace element concentrations in feed samples (mean, minimum - maximum) referred to 88% DM.

Feed sample	N	Ca (g/kg)	P (g/kg)	Ca:P ratio	Zn (mg/kg)	Mn (mg/kg)	Se (mg/kg)
Starter feed	11	14.39 (10.65 - 24.83)	9.63 (8.10 - 15.98)	1.49 (1.31 - 1.70)	153.93 (125.85 - 303.48)	141.56 (108.55 - 278.75)	0.91 (0.32 - 1.59)
Phase 1	11	13.29 (11.57 - 16.87)	8.73 (7.44 - 10.12)	1.53 (1.26 - 1.73)	137.56 (125.99 -158.67)	113.88 (83.15 - 141.65)	0.93 (0.32 - 1.32)
Phase 2	10	12.15 (10.58 - 15.31)	7.76 (7.04 - 8.53)	1.56 (1.35 - 1.81)	128.39 (117.66 - 146.83)	110.41 (82.13 - 127.10)	0.71 (0.40 - 0.99)
Phase 3	10	11.01 (10.04 - 12.34)	6.97 (6.47 - 7.51)	1.58 (1.45 - 1.91)	126.84 (105.13 - 145.68)	108.26 (86.73 - 138.08)	0.71 (0.33 - 0.99)
Final fattening feed	10	10.98 (7.42 - 12.50)	6.82 (6.32 - 7.01)	1.61 (1.17 - 1.82)	120.80 (91.58 - 133.81)	111.72 (79.25 - 152.1)	0.63 (0.34 - 0.92)
Oat	5	1.02 (0.54 - 1.55)	3.54 (3.22 - 3.76)	0.29 (0.15 - 0.41)	22.88 (17.19 - 29.77)	25.07 (12.33 - 33.77)	0.08 (0.01 - 0.178)
Wheat	1	0.99	2.79	0.35	21.90	23.08	0.05

Ca: calcium; DM: dry matter; Mn: manganese; P: phosphorus; Se: selenium; Zn: zinc.

Table S7. Vitamin concentrations in feed samples (mean, minimum - maximum) referred to 88% DM.

Feed sample	N	Vitamin D_3 (IE/kg)	Vitamin E (mg/kg)
Starter feed	11	3858.00 (2742.00 - 5841.00)	52.09 (41.36 - 109.41)
Phase 1	11	3340.45 (2272.00 - 4240.00)	46.82 (40.94 - 56.30)
Phase 2	10	3472.80 (2200.00 - 4371.00)	45.75 (32.95 - 54.82)
Phase 3	10	3632.50 (2059.00 - 4420.00)	47.59 (40.78 - 51.87)
Final fattening feed	10	3673.10 (2914.00 - 4335.00)	44.26 (38.86 - 50.16)
Oat	5	< 880	10.68 (7.67 - 12.92)
Wheat	1	< 880	12.81

DM: dry matter.

Table S8. Raw nutrient and energy concentrations in feed samples: Actual supply during recommendation periods of the Society of Nutrition Physiology [31] referred to 88% DM or MJ AMEn; A = mean (minimum - maximum), B = recommendation [31,53] [1].

Phase		N	CP (g/kg)	AMEn (kcal/kg)	AMEn (MJ/kg)	CP (g/MJ)[1]	CF (g/kg)[1]
0	A	10	278.33 (261.42 - 292.16)	2,720.45 (2,672.69 – 2,773.00)	11.39 (11.19 - 11.61)	24.45 (22.80 - 25.80)	37.61 (29.71 - 43.22)
	B		253.00		11.20 - 11.70	22.20	35.00
1	A	10	265.24 (249.27 - 284.99)	2,732.40 (2,665.52 – 2,782.55)	11.44 (11.16 - 11.65)	23.57 (21.58 - 24.95)	36.93 (31.1 - 42.4)
	B		238.00		11.50 - 12.00	20.50	35.00
2	A	10	243.97 (214.73 - 261.09)	2,773.00 (2,708.51 – 2,858.99)	11.61 (11.34 - 11.97)	21.20 (18.25 - 23.05)	40.75 (35.42 - 52.54)
	B		237.00		11.80 - 12.30	20.40	40.00 – 45.00
3	A	10	188.11 (170.43 - 203.26)	2,796.89 (2,744.34 – 2,844.65)	11.71 (11.49 - 11.91)	16.93 (14.59 - 20.17)	41.08 (36.18 - 50.80)
	B		210.00		12.30 - 12.80	17.50	40.00 – 45.00
4	A	10	162.66 (153.09 - 176.56)	2,794.50 (2,694.18 – 2,839.88)	11.70 (11.28 - 11.89)	14.95 (13.00 - 24.31)	39.15 (36.28 - 42.91)
	B		154.00		12.80 - 13.20	12.50	40.00 – 45.00
5	A	10	155.18 (145.85 - 166.78)	2,794.50 (2,694.18 – 2,873.32)	11.70 (11.28 - 12.03)	14.25 (12.31 - 23.48)	38.32 (34.73 - 41.78)
	B		-		12.80 - 13.20	-	-

AMEn: apparent metabolizable energy corrected by nitrogen; CF: crude fiber; CP: crude protein, DM: dry matter.

Table S9. Amino acid concentrations in feed samples: Actual supply during recommendation periods of the Society of Nutrition Physiology [31] referred to 88% DM or MJ AMEn; A = mean (minimum - maximum), B = recommendation [31,53] [1].

Phase		N	THR (g/kg)	THR (g/MJ AMEn)[1]	MET (g/kg)	MET (g/MJ AMEn)[1]	MET + CYS (g/kg)	MET + CYS (g/MJ AMEn)[1]	LYS (g/kg)
0	A	10	11.24 (9.85 - 13.86)	0.99 (0.85 - 1.21)	5.28 (4.56 - 5.97)	0.46 (0.39 - 0.52)	9.60 (8.69 - 10.38)	0.84 (0.75 - 0.91)	14.66 (13.82 - 15.65)
	B		9.30	0.86	5.70	0.48	8.40	0.76	17.00
1	A	10	10.49 (9.44 - 11.59)	0.92 (0.82 - 1.02)	5.08 (3.84 - 5.80)	0.44 (0.33 - 0.50)	9.28 (7.79 - 10.96)	0.81 (0.67 - 0.95)	13.93 (12.66 - 15.13)
	B		9.00	0.79	5.60	0.44	8.50	0.71	16.20
2	A	10	9.31 (7.82 - 10.42)	0.80 (0.67 - 0.92)	4.35 (3.69 - 5.03)	0.37 (0.32 - 0.44)	8.29 (6.93 - 9.57)	0.72 (0.61 - 0.83)	12.57 (11.73 - 13.43)
	B		8.60	0.78	5.50	0.41	8.40	0.68	15.50
3	A	10	7.13 (6.19 - 7.81)	0.61 (0.53 - 0.67)	3.43 (3.07 - 4.33)	0.32 (0.26 - 0.56)	6.79 (6.06 - 7.74)	0.58 (0.51 - 0.66)	9.61 (8.21 - 10.69)
	B		8.20	0.74	5.20	0.39	8.20	0.67	14.60
4	A	10	5.87 (5.20 - 6.77)	0.50 (0.44 - 0.58)	2.67 (2.02 - 3.80)	0.43 (0.17 - 2.23)	5.67 (4.99 - 6.97)	0.48 (0.42 - 0.60)	8.10 (7.24 - 9.77)
	B		6.30	0.58	3.90	0.32	6.40	0.56	11.40
5	A	10	5.48 (4.65 - 5.90)	0.47 (0.39 - 0.49)	2.41 (1.92 - 3.27)	0.41 (0.16 - 2.23)	5.29 (4.44 - 6.25)	0.45 (0.38 - 0.54)	7.55 (6.82 - 8.68)

AMEn: apparent metabolizable energy corrected by nitrogen; CYS: cysteine; DM: dry matter; LYS: lysine; MET: methionine; THR: threonine.

Table S10. Amino acid concentrations in feed samples: Actual supply during recommendation periods of the Society of Nutrition Physiology [31] referred to 88% DM or MJ AMEn; A = mean (minimum - maximum), B = recommendation [31,53] [1].

Phase		N	LYS (g/MJ AMEn)[1]	ARG (g/kg)	VAL (g/kg)	ISO (g/kg)	LEU (g/kg)	PHE (g/kg)	HIS (g/kg)
0	A	10	1.29 (1.21 - 1.37)	16.49 (15.27 - 17.46)	14.02 (13.07 - 15.53)	11.89 (11.03 - 12.76)	25.53 (23.41 - 28.7)	14.12 (13.14 - 15.61)	6.30 (5.90 - 6.80)
	B		1.41	14.90	11.10	9.40	18.20	10.50	5.50
1	A	10	1.22 (1.09 - 1.33)	16.04 (15.17 - 16.86)	13.50 (12.15 - 15.35)	11.37 (10.70 - 12.48)	24.80 (21.64 - 28.38)	13.58 (12.19 - 15.28)	6.12 (5.75 - 6.52)
	B		1.36	14.60	10.80	9.10	17.30	9.90	5.60
2	A	10	1.31 (1.00 - 3.42)	14.93 (13.75 - 15.91)	12.08 (10.61 - 13.02)	10.24 (8.96 - 11.07)	21.48 (16.76 - 23.58)	11.56 (9.02 - 13.42)	5.55 (5.07 - 5.94)
	B		1.29	14.20	10.40	8.90	16.50	9.30	5.50
3	A	10	0.82 (0.71 - 0.90)	12.17 (10.91 - 13.22)	9.38 (8.47 - 10.18)	7.88 (6.89 - 8.75)	15.61 (12.85 - 20.78)	9.07 (8.11 - 10.18)	4.45 (4.04 - 4.84)
	B		1.16	13.50	9.80	8.60	15.60	8.50	5.40
4	A	10	0.69 (0.61 - 0.82)	10.92 (10.27 - 11.99)	7.95 (7.48 - 9.17)	6.66 (6.20 - 7.69)	12.38 (11.43 - 14.4)	7.82 (7.30 - 9.02)	3.95 (3.78 - 4.37)
	B		0.88	10.40	7.60	6.80	12.10	6.40	4.20
5	A	10	0.65 (0.58 - 0.75)	10.46 (9.80 - 11.92)	7.39 (6.87 - 8.17)	6.20 (5.73 - 6.91)	11.39 (10.51 - 12.50)	7.32 (6.73 - 8.20)	3.76 (3.55 - 4.10)

ARG: arginine; DM: dry matter; HIS: histidine; ISO: isoleucine; LEU: leucine; LYS: lysine; PHE: phenylalanine; VAL: valine.

Table S11. Bulk and trace element concentrations in feed samples: Actual supply during recommendation periods of the Society of Nutrition Physiology [31] referred to 88% DM; A = mean (minimum - maximum), B = recommendation [31].

Phase		N	Ca (g/kg)	P (g/kg)	Ca:P ratio	Zn (mg/kg)	Mn (mg/kg)	Se (mg/kg)
0	A	10	13.97 (11.02 - 17.47)	9.20 (8.11 - 11.02)	1.52 (1.36 - 1.69)	144.39 (125.85 – 196.28)	129.55 (100.39 – 170.73)	0.92 (0.32 - 1.15)
	B		13.00	10.00	1.30*	44.00	52.80	0.18
1	A	10	13.22 (10.98 - 16.42)	8.70 (7.77 - 9.67)	1.53 (1.28 - 1.71)	135.52 (130.05 - 148.90)	115.39 (83.15 - 144.25)	0.89 (0.36 - 1.27)
	B		13.00	10.00	1.30*	44.00	52.80	0.18
2	A	10	12.39 (11.03 - 15.51)	8.03 (7.48 - 8.73)	1.55 (1.36 - 1.78)	131.90 (122.73 - 144.02)	112.21 (88.88 - 134.89)	0.75 (0.37 - 1.09)
	B		11.00	9.00	1.22*	35.20	44.00	0.18
3	A	10	11.35 (10.10 - 13.16)	7.13 (6.65 - 7.91)	1.59 (1.45 - 1.87)	126.72 (105.13 - 144.00)	109.18 (86.73 - 128.38)	0.72 (0.36 - 0.96)
	B		9.00	6.00	1.50*	35.20	44.00	0.18
4	A	10	11.29 (10.19 - 12.50)	6.86 (6.42 - 7.33)	1.64 (1.51 - 1.82)	124.71 (112.38 - 133.81)	113.45 (93.36 - 152.10)	0.64 (0.33 - 0.88)
	B		7.00	5.00	1.40*	35.20	44.00	0.18
5	A	10	11.04 (7.42 - 12.5)	6.82 (6.32 - 7.01)	1.61 (1.17 - 1.82)	120.45 (91.58 - 133.81)	111.72 (79.25 - 152.10)	0.63 (0.34 - 0.92)
	B		-	-	-	35.20	44.00	0.18

* calculated from the recommended Ca and P concentrations (GfE, 2004); Ca: calcium; DM: dry matter; Mn: manganese; P: phosphorus; Se: selenium; Zn: zinc.

Table S12. Vitamin concentrations in feed samples: Actual supply during recommendation periods of the Society of Nutrition Physiology [31] referred to 88% DM; A = mean (minimum - maximum), B = recommendation [31].

Phase		N	Vitamin D_3 (IE/kg)	Vitamin E (mg/kg)
0	A	10	3,550.53 (2,854.18 – 4,542.79)	48.73 (41.07 - 68.97)
	B		1,320.00	13.20
1	A	10	3,343.68 (2,272.00 – 4,273.64)	45.22 (40.94 - 49.06)
	B		968.00	13.20
2	A	10	3,494.18 (2,215.38 – 4,285.82)	45.96 (34.35 - 53.13)
	B		968.00	13.20
3	A	10	3,539.70 (2,518.41 – 4,192.86)	47.13 (38.27 - 51.69)
	B		968.00	8.80
4	A	10	3526.01 (2,385.73 – 4,066.43)	45.67 (40.47 - 50.16)
	B		968.00	8.80
5	A	10	3,673.10 (2,914.00 – 4,732.00)	44.26 (38.86 - 50.16)
	B		968.00	8.80

DM: dry matter.
